# 3-(3-Pyridylmethylidene)-2-indolinone Reduces the Severity of Colonic Injury in a Murine Model of Experimental Colitis

**DOI:** 10.1155/2015/959253

**Published:** 2015-03-22

**Authors:** Kun-Ping Wang, Chao Zhang, Shou-Guo Zhang, En-Dong Liu, Lan Dong, Xiang-Zhen Kong, Peng Cao, Chun-Ping Hu, Ke Zhao, Yi-Qun Zhan, Xiao-Ming Dong, Chang-Hui Ge, Miao Yu, Hui Chen, Lin Wang, Xiao-Ming Yang, Chang-Yan Li

**Affiliations:** ^1^State Key Laboratory of Proteomics, Beijing Proteome Research Center, Beijing Institute of Radiation Medicine, Beijing 100850, China; ^2^Anhui Medical University, Hefei 230032, China; ^3^Department of Anesthesiology, General Hospital of Chinese People's Armed Police Forces, Beijing 100039, China; ^4^School of Chemical Engineering and Technology, Department of Pharmaceutical Engineering, Tianjin University, Tianjin 300072, China; ^5^Laboratory of Cellular and Molecular Biology, Jiangsu Province Institute of Traditional Chinese Medicine, No. 100, Shizi Street, Hongshan Road, Nanjing, Jiangsu 210028, China

## Abstract

Nrf2 is the key transcription factor regulating the antioxidant response which is crucial for cytoprotection against extracellular stresses. Numerous *in vivo* studies indicate that Nrf2 plays a protective role in anti-inflammatory response. 3-(3-Pyridylmethylidene)-2-indolinone (PMID) is a synthesized derivative of 2-indolinone compounds. Our previous study suggested that PMID induces the activation of Nrf2/ARE pathway, then protecting against oxidative stress-mediated cell death. However, little is known regarding the anti-inflammatory properties of PMID in severe inflammatory phenotypes. In the present study we determined if PMID treatment protects mice from dextran sodium sulphate- (DSS-) induced colitis. The result suggests that treatment with PMID prior to colitis induction significantly reduced body weight loss, shortened colon length, and decreased disease activity index compared to control mice. Histopathological analysis of the colon revealed attenuated inflammation in PMID pretreated animals. The levels of inflammatory markers in colon tissue and serum were reduced associated with inhibition of NF-*κ*B activation. The expression levels of Nrf2-dependent genes such as HO-1, NQO1, and Nrf2 were increased in PMID pretreated mice. However, PMID pretreatment did not prevent DSS-induced colitis in Nrf2 knockout mice. These data indicate that PMID pretreatment in mice confers protection against DSS-induced colitis in Nrf2-dependent manner, suggesting a potential role of PMID in anti-inflammatory response.

## 1. Introduction

Chronic inflammation has been identified as a potential risk factor for colorectal and other cancers. Inflammatory bowel diseases (IBD) are forms of chronic, recurrent colitis, most commonly Crohn's disease and ulcerative colitis, and many epidemiologic and clinical studies have shown that IBD increases the risk of colorectal cancer [1]. Reactive oxygen and nitrogen species are thought to be a major factor underlying the contribution of chronic inflammation to neoplastic transformation [2].

Nrf2 is the key transcription factor regulating the antioxidant response which is crucial for cytoprotection against extracellular stresses. Upon oxidative or electrophilic insults, Nrf2 will translocate into the nucleus where it binds with antioxidant response elements and transactivates phase II detoxifying and antioxidant genes [3]. Numerous* in vivo* studies indicate that Nrf2 may play a role in the regulation of inflammation. Nrf2 protects against chemical-induced pulmonary injury and inflammation [4–6], whereas genetic ablation of Nrf2 enhances the susceptibility to cigarette smoke-induced emphysema and to severe airway inflammation and asthma in mice [6, 7]. In addition, Nrf2 was found to be a crucial regulator of the innate immune response and survival during experimental sepsis [8]. Moreover, disruption of Nrf2 gene renders animals more susceptible to dextran sodium sulphate- (DSS-) induced colitis and to AOM-DSS-induced colon carcinogenesis [9–11]. Therefore, Nrf2 pathway appears to mediate a strong anti-inflammatory response, besides induction of detoxification and antioxidant enzymes.

Oxidative and inflammatory injuries are closely linked to each other in the process of multistage carcinogenesis. Thus, compounds with anti-inflammatory activities are anticipated to inhibit oxidative stress, and vice versa. 3-(3-Pyridylmethylidene)-2-indolinone (PMID) is a synthesized derivative of 2-indolinone compounds. Our previous study suggested that PMID induces the ARE-mediated genes expression through stabilization of Nrf2 protein and activation of Nrf2/ARE pathway and protects against oxidative stress-mediated cell death, indicating that PMID is a novel antioxidant agent by triggering the induction of antioxidant and defensive genes [12]. However, little is known regarding the anti-inflammatory properties of PMID in severe inflammatory phenotypes. In the present study we determined if PMID treatment protects mice from dextran sodium sulphate- (DSS-) induced colitis.

## 2. Materials and Methods

### 2.1. Animals

Nrf2^−/−^ (C57BL/6J) mice were purchased from The Jackson Laboratory (USA). Nrf2^+/+^ (C57BL/6J) mice and adult male ICR mice were obtained from the Beijing Institute of Radiation Medicine (BIRM) Animal Center (Beijing, China). All the mice were housed in a climate-controlled, circadian rhythm-adjusted room and allowed food and water ad libitum. The animals were, on average, 6 to 8 weeks of age and weighed between 30 and 34 g at the time of experiment. PMID was dissolved in 0.5% CMC-Na as a 10 *μ*mol/mL stock solution. Mice were gavaged with 2.2, 11, or 22 mg PMID/kg body weight using a vehicle of 0.2 mL 0.5% CMC-Na once daily for one week. Model control (MC) mice were gavaged with 0.5% CMC-Na only. All injections were administered orally by gavage using a sonde. All treatment procedures were approved by the Animal Care Committee of BIRM. Animals received humane care according to the criteria outlined in the “Guide for the Care and Use of Laboratory Animals” prepared by the National Academy of Sciences and published by the National Institutes of Health.

### 2.2. Colitis Induction and Determination of Clinical Scores in ICR Mice

Acute colitis was induced by the application of 3% DSS via the drinking water for 7 days. During DSS treatment, body weight, stool consistency, and the presence of blood were examined daily, the combined scores of which are summarized as the disease activity index (DAI) provided in Supplementary Table S1 available online at http://dx.doi.org/10.1155/2015/959253. DAI = (weight loss + stool consistency + stool blood)/3 [13, 14].

### 2.3. Histopathological Analysis

At the end of the experiment (1 week DSS exposure in drinking water), all the mice were sacrificed. For histopathological analysis, 1 cm distal colon samples were fixed in 4% paraformaldehyde, sectioned, and stained with hematoxylin/eosin. Colon images were captured under 100x magnification.

### 2.4. RNA Isolation and Real-Time Polymerase Chain Reaction (PCR)

Total RNA isolation and reverse-transcription were applied according to the manufacture's protocol (Promega Corp., Madison, WI, USA). The cDNA was analyzed using real-time PCR with SYBR Green Real-Time PCR Master Mix (TOYOBO, Osaka, Japan). The level of GAPDH mRNA was used as an internal standard. Differential expression was calculated according to the 2^−ΔΔCT^ method. The abundance of mRNA of each gene was normalized to GAPDH. The sequences of the primers are provided in Supplementary Table S2.

### 2.5. Cytokine Analysis

For cytokine analysis, orbital blood was isolated from the mice for 400 *μ*L, followed by centrifugation at 3500 rmp × 15 min; the supernatant was detected (Mouse Inflammation Kit, BD, USA) in a BD FACSCalibur. Protein extracts from mouse tissues were determined by ELISA (Boster Bio-Engineering Co., Wuhan).

### 2.6. Western Blot Analysis

Cell extracts were prepared in RIPA buffer (Beyotime, China). Nuclear extracts were prepared with a Nuclear Extract Kit (Thermo Scientific, USA) according to the manufacturer's recommendations. Then, Western blot analysis was performed according to standard procedures. Antibodies were used at the following concentrations: Nrf2 (Abcam), 1 : 1000; HO-1 (Santa Cruz), 1 : 500; c-jun (Santa Cruz), 1 : 1000; Histone H2B (Abcam), 1 : 1000; GAPDH (Santa Cruz), 1 : 1000; p65 (Santa Cruz), 1 : 1000; c-Jun (Santa Cruz), 1 : 1000.

### 2.7. NF-*κ*B Assay

For measurement of NF-*κ*B activation, nuclear extracts were isolated from snap frozen mouse colonic tissues using a Nuclear Extract Kit (Thermo Scientific, USA) according to the manufacturer's instructions. After measurement of protein concentration by the Bradford method, 1 *μ*g of nuclear extract was used to measure the NF-*κ*B activity using Thermo Scientific NF-*κ*B p65 Transcription Factor Kit (Thermo Scientific, USA) according to the manufacturer's instructions. Chemiluminescent intensities were calculated as relative light units (RLU) and normalized with the mean RLU from untreated animals.

### 2.8. Statistical Analysis

Differences between the groups were determined utilizing GraphPad Prism 5 software. Data were reported as mean ± SD and the statistical significance was assessed by one-way analysis of variance (ANOVA) with a Kruskal-Wallis test and Newman-Keuls posttest. A value of *P* ≤ 0.05 was considered to be significant.

## 3. Results

### 3.1. PMID Pretreatment Attenuates DSS-Induced Colitis in ICR Mice

**Figure 1:**
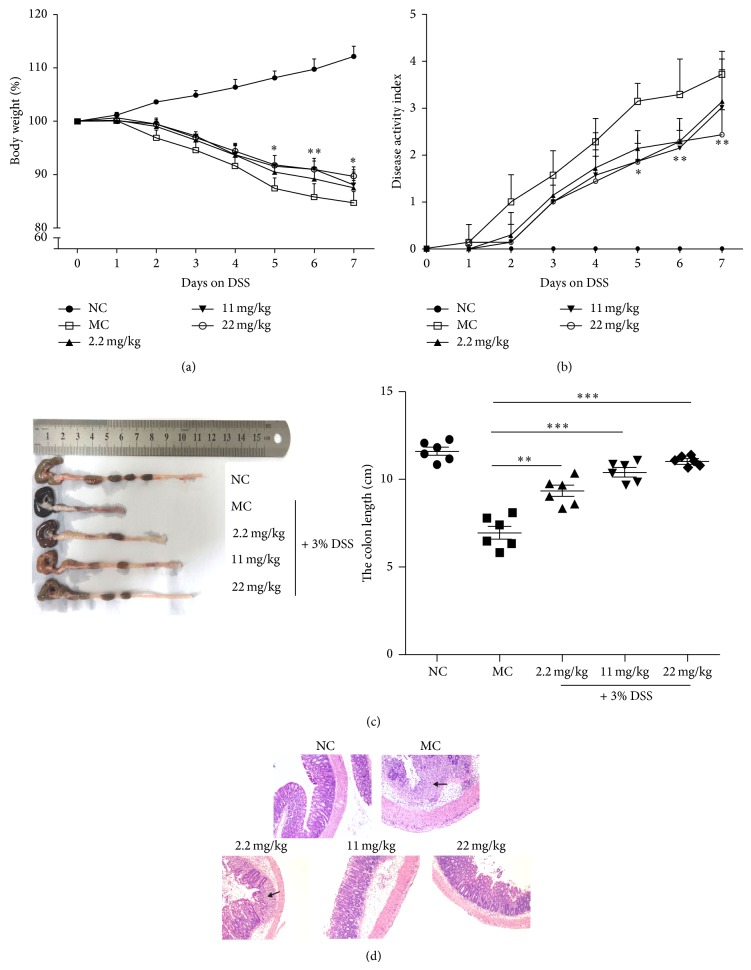
PMID pretreatment attenuates DSS-induced colitis in ICR mice. Following treatment with the indicated doses of PMID* per os* for 7 days, colitis was induced by DSS (3%) via drinking water for 7 days. During DSS exposure weight loss of the animals was recorded daily (a). Mice without DSS and PMID treatment were used as normal control (NC) and mice treated with DSS only without PMID were used as model control (MC). DAI incorporating BW loss, diarrhoea, and rectal bleeding score was assessed daily (b) (*n* = 10, data are expressed as mean ± SD). Following sacrifice, colons length was measured (c) and then colon samples were fixed in 4% paraformaldehyde, stained with hematoxylin/eosin, and visualized at 10x magnification (d). Representative images of severely inflammatory cell infiltration (arrowheads) and loss of colonic crypts in MC group compared to NC group. Results represented mean ± SD. *n* = 10/group. The statistical difference between the samples was demonstrated as ^*^ *P* ≤ 0.05 or ^**^ *P* ≤ 0.01 or ^***^ *P* ≤ 0.001.

Mice that received 2.2, 11, or 22 mg PMID/kg body weight (BW)* per os* for 7 days prior to colitis induction by 3% DSS developed less severe symptoms of colitis in comparison to model control (MC) mice. Mice without any treatment were used as normal control (NC). Weight loss during DSS treatment was significantly lower in animals pretreated with PMID compared to those given 0.5% CMC-Na (Figure 1(a)). Furthermore, DAI (denoting a combined score of weight loss, diarrhoea, and rectal bleeding) was also significantly lower in PMID pretreated animals (Figure 1(b)). Compared to normal control, the model control mice showed reduced length of colon, while the colons of PMID pretreated mice were shortened to a greater extent (Figure 1(c)). Microscopic analysis of colon tissue also showed that colitis mice pretreated with PMID had noticeably lower levels of inflammatory cell infiltration into their distal colon mucosa, loss of colonic crypts, and epithelial cell necrosis compared to MC mice (Figure 1(d)). When PMID treated mice alone, no effect was observed on the colon length, colorectal epithelia, and inflammation (data not shown). These results suggest that PMID pretreatment attenuates the severity of DSS-induced colitis.

### 3.2. PMID Pretreatment Lessens Proinflammatory Biomarkers in Serum of DSS-Induced ICR Mice

**Figure 2:**
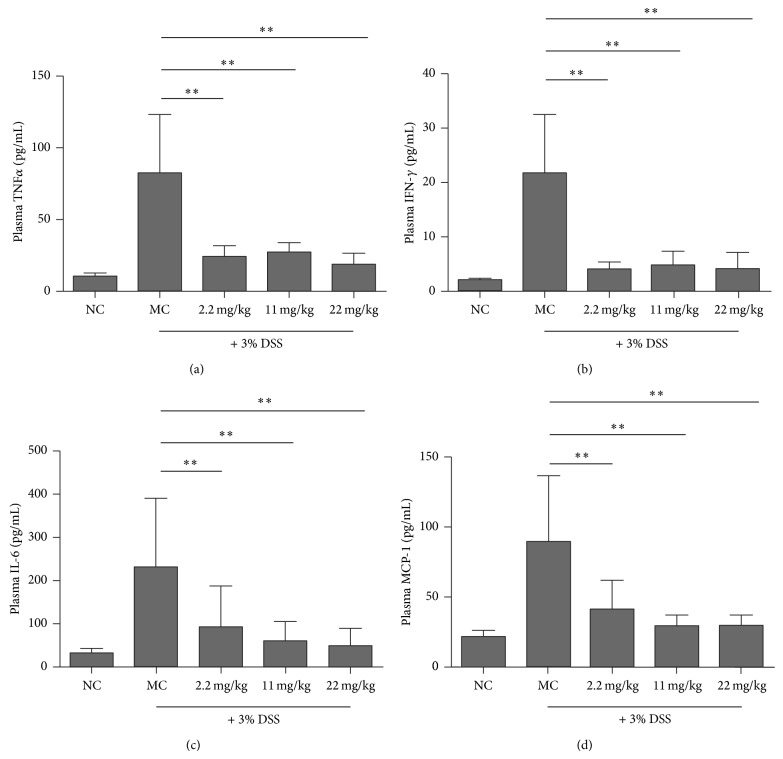
PMID pretreatment attenuated the levels of proinflammatory markers in serum of DSS-induced ICR mice. Following treatment with the indicated doses of PMID* per os* for 7 days, colitis was induced by DSS (3%) via drinking water for 7 days. Then the serum was collected and the protein levels of TNF*α*, IL-6, IFN-*γ*, and MCP-1 were analyzed using CBA just as “Section 2” described. Results represented mean ± SD. *n* = 10/group. The statistical difference between the samples was demonstrated as ^*^ *P* ≤ 0.05 or ^**^ *P* ≤ 0.01.

The levels of various proinflammatory biomarkers, IL-6, TNF*α*, MCP-1, and IFN-*γ*, in serum of DSS-induced ICR mice were analyzed using ELISA. As shown in Figure 2, DSS treatment led to increased serum levels of IL-6, TNF*α*, MCP-1, and IFN-*γ*, and PMID pretreatment significantly lessens the levels of these biomarkers.

### 3.3. PMID Pretreatment Lessens Proinflammatory Biomarkers in Colon Tissue of DSS-Induced ICR Mice

**Figure 3:**
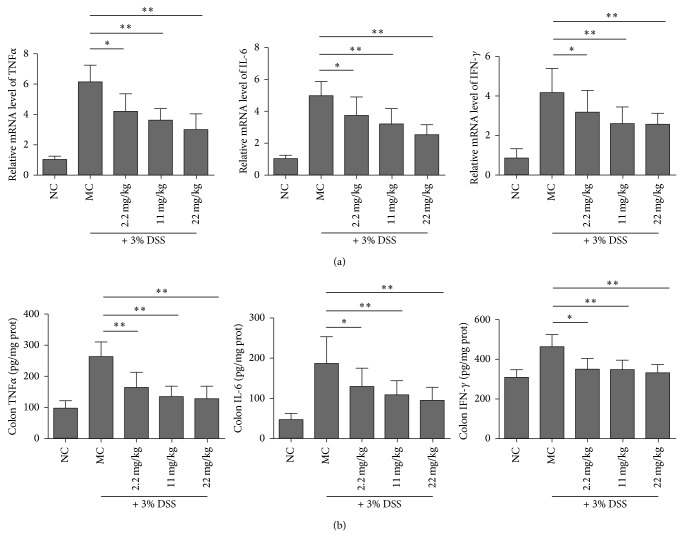
PMID pretreatment lessens expression levels of proinflammatory markers in colon tissue of DSS-induced ICR mice. Following treatment with the indicated doses of PMID* per os* for 7 days, colitis was induced by DSS (3%) via drinking water for 7 days. Then the mice were sacrificed and the colons were excised. Total RNA was extracted and the mRNA levels of the genes indicated were analyzed using real-time PCR. The relative expression levels of genes in normal control (NC) mice were set as 1. The data were normalized to GAPDH expression. (b) Cell lysates were prepared and the protein levels of the indicated genes were measured using ELISA. Results represented mean ± SD. *n* = 10/group. The statistical difference between the samples was demonstrated as ^*^ *P* ≤ 0.05 or ^**^ *P* ≤ 0.01.

Furthermore, the mRNA levels of TNF*α*, IFN-*γ*, and IL-6 in the colons of the mice exposed to DSS were analyzed using real-time PCR (Figure 3). Consistent with the result from serum, the mRNA levels of these genes in colon tissue were upregulated after DSS treatment, and PMID pretreatment resulted in a significant downregulation of the mRNA level of these biomarkers compared to model control (Figure 3(a)). The protein levels of these biomarkers were measured using ELISA and the similar results were obtained (Figure 3(b)). These results suggest that PMID pretreatment lessens proinflammatory biomarkers in colon tissue of DSS-induced ICR mice.

### 3.4. NF-*κ*B Activation Is Decreased in Colon Tissue of DSS-Induced ICR Mice with PMID Pretreatment

**Figure 4:**
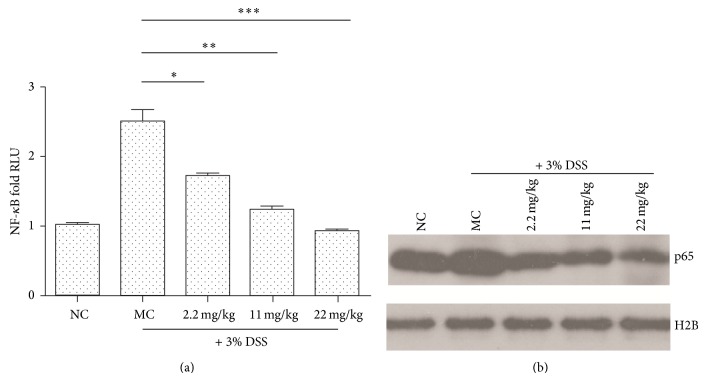
NF-*κ*B activation is decreased in colon tissue of DSS-induced ICR mice with PMID pretreatment. Mice were treated with the indicated doses of PMID* per os* for 7 days and then induced to colitis by DSS via drinking water for 7 days. Then the mice were sacrificed and nuclear extracts were prepared for NF-*κ*B activity assay just as “Section 2” described (a). The RLU was normalized with the mean RLU from normal control group. Results represented mean ± SD. *n* = 3/group. The statistical difference between the samples was demonstrated as ^*^ *P* ≤ 0.05 or ^**^ *P* ≤ 0.01. (b) The p65 protein level in nuclear extracts was measured by Western blotting. Histone H2B was used as internal control.

The link between inflammation and NF-*κ*B signaling pathway has been widely reported and the transcription of inflammatory cytokines is largely mediated by the NF-*κ*B. Thus, we examined the NF-*κ*B activation using a chemiluminescence-based assay kit after 7 days of DSS treatment. As shown in Figure 4(a), DSS treatment led to significant increase of NF-*κ*B activity, and PMID pretreatment attenuated the activation level of NF-*κ*B. Furthermore, we investigated the p65 protein level in nuclear extracts (Figure 4(b)), and the similar results were obtained. These data highlight that, with decreased inflammatory response in PMID pretreatment group, the NF-*κ*B signaling pathway activation was also inhibited.

### 3.5. PMID Induces the Nrf2/ARE Pathway Activation in DSS-Treated ICR Mice

**Figure 5:**
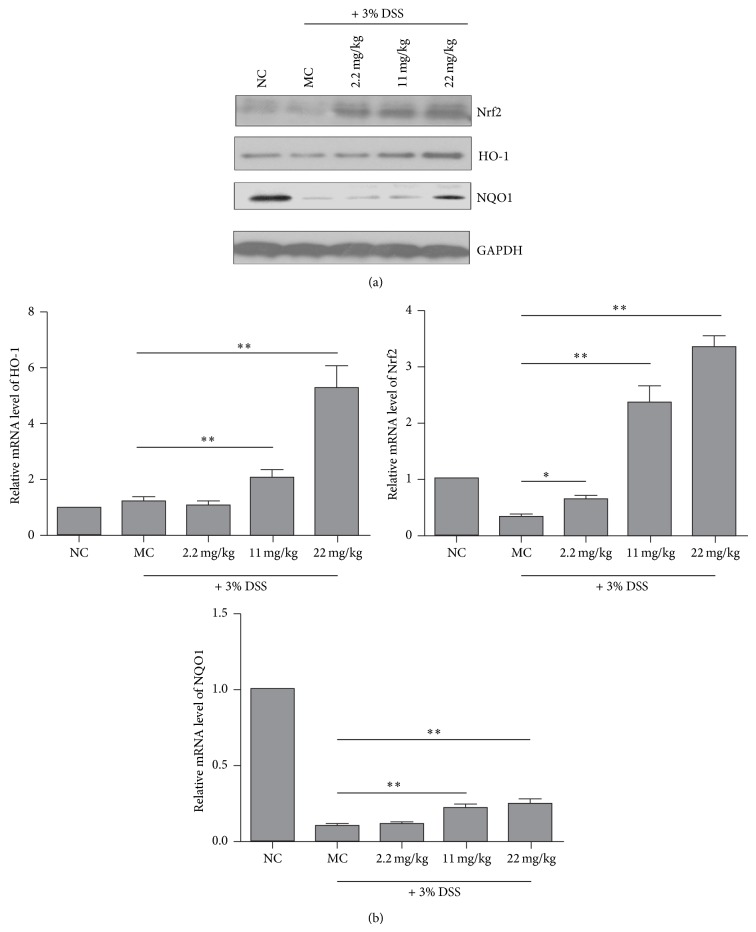
PMID induces the Nrf2/ARE pathway activation in DSS-treated ICR mice. Mice were treated with the indicated doses of PMID* per os* for 7 days and then induced to colitis by DSS via drinking water for 7 days. Then the mice were sacrificed and total cell lysates were prepared for analyzing the protein levels of Nrf2, HO-1, and NQO1 (a). GAPDH was used as internal control. Total RNA was extracted for analyzing the mRNA levels of the genes indicated using real-time PCR (b). The relative expression levels in normal control (NC) group mice were set as 1. The data were normalized to GAPDH expression. Each bar represented the mean ± SD for 7 mice of each group. The statistical difference between the samples was demonstrated as ^*^ *P* ≤ 0.05 or ^**^ *P* ≤ 0.01.

Our previous study suggested that PMID induces Nrf2/ARE pathway through accelerating Nrf2 protein accumulation. Therefore, we investigated the Nrf2 protein level after PMID and DSS treatment. As shown in Figure 5(a), DSS treatment led to downregulation of Nrf2 protein level and PMID pretreatment significantly increased Nrf2 protein level, which was consistent with our previous study. We further detected the mRNA levels of Nrf2 target genes including NAPDH-quinone oxidoreductase-1 (NQO1), heme oxygenase-1 (HO-1), and Nrf2 in DSS-treated mice. As shown in Figure 5(b), DSS treatment led to downregulation of Nrf2 and NQO1, but the levels of HO-1 did not changed significantly, which was consistent with previous study [9]. In PMID pretreated mice, the expression levels of NQO1, HO-1, and Nrf2 were elevated significantly in a dose-dependent manner compared to model control group. With the increased Nrf2 protein accumulation, the protein levels of HO-1 and NQO1 were also increased (Figure 5(a)).

In addition to Nrf2, the transcription factor AP-1 binds to some ARE sites and activates transcription of antioxidant/phase II detoxifying enzymes, including HO-1 and NQO-1 [15]. AP-1 is a dimeric transcription factor composed of c-Jun, c-Fos, or activating transcription factor subunits. We investigated the protein level of c-Jun in nuclear extracts of colon tissue. As shown in Supplementary Figure 1, PMID treatment did not affect the level of AP-1.

These results suggest that PMID induces Nrf2/ARE pathway in DSS-treated ICR mice.

### 3.6. PMID Prevents DSS-Induced Colitis in Nrf2-Dependent Manner

**Figure 6:**
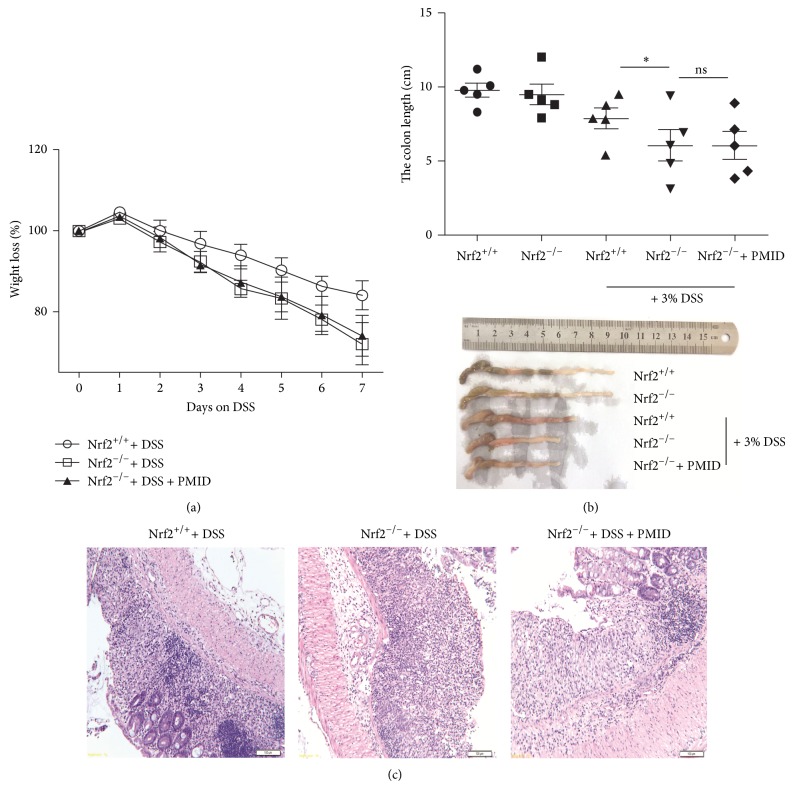
PMID prevents DSS-induced colitis in Nrf2-dependent manner. Nrf2^−/−^ mice were received 22 mg PMID/kg BW* per os* for 7 days prior to colitis induction by 3% DSS and the weight loss was measured. Nrf2^+/+^ mice were used as control. During DSS exposure weight loss of the animals was recorded daily (a). Following sacrifice, colons length was measured (b) and then colon samples were fixed in 4% paraformaldehyde, stained with hematoxylin/eosin, and visualized at 10x magnification (c). The statistical difference between the samples was demonstrated as ^*^ *P* ≤ 0.05.

To further investigate if PMID prevents DSS-induced colitis via Nrf2/ARE pathway, the effect of PMID on DSS-induced Nrf2^−/−^ mice was measured. Nrf2^−/−^ mice were received 22 mg PMID/kg BW* per os* for 7 days prior to colitis induction by 3% DSS and the weight loss was measured. Nrf2^+/+^ mice were used as control. As shown in Figure 6, Nrf2^−/−^ mice showed increased sensitivity to DSS-induced colitis compared to Nrf2^+/+^ mice with higher weight loss (Figure 6(a)), shortened colon length (Figure 6(b)), and elevated level of inflammatory cell infiltration with destroyed colon structure (Figure 6(c)). However, no difference was observed between Nrf2^−/−^ mice and PMID pretreated Nrf2^−/−^ mice. These data suggest that PMID prevents DSS-induced colitis in Nrf2-dependent manner.

## 4. Discussion

Increased free radicals and impaired antioxidant defenses in the intestines have been linked to the pathogenesis of inflammatory bowel diseases (IBD) [16]. The dextran sulfate sodium- (DSS-) induced mouse model of colitis is one of the most widely used models that mimics ulcerative colitis-like disease in humans [17]. This model system has been used to reveal important events leading to IBD and colorectal carcinogenesis. It was suggested that Nrf2 plays an important role in protecting intestinal integrity via regulation of proinflammatory cytokines and induction of phase II detoxifying enzymes [9], indicating that Nrf2 may serve as novel target for designing therapies to prevent and treat inflammatory bowel diseases such as Crohn's disease and ulcerative colitis. In the present study, we showed that pretreatment with an inducer of Nrf2 pathway named PMID significantly attenuates symptoms of DSS-induced colitis including DAI, body weight, colon length, and histology. The levels of inflammatory markers in colon tissue and serum were reduced associated with reduced activation of NF-*κ*B pathway. Furthermore, the expression levels of Nrf2 target genes such as HO-1, NQO1, and Nrf2 were increased in PMID pretreated mice. Our results provide the first line of evidence that PMID confers protection from DSS-induced colitis in mice, suggesting a potential role of PMID in anti-inflammatory response.

The protective role of Nrf2 activation has also been established in many human disorders including cancer, Alzheimer's and Parkinson's diseases, chronic obstructive pulmonary disease (COPD), asthma, atherosclerosis, diabetes, IBD, multiple sclerosis, osteoarthritis, and rheumatoid arthritis [18]. Regulation of Nrf2-ARE signaling has also been implicated in the determination of health span, longevity, and aging [19]. The emerging role of Nrf2 pathway in oxidative stress-related pathologies offers novel therapeutic opportunities. Pharmacological interventions are being actively pursued for the discovery of modulators of this pathway as potential preventive and therapeutic agents. In recent years, research has been highly focused toward the discovery of new Nrf2-related drugs. Andrographolide possesses antioxidative properties against cigarette smoke-induced lung injury via augmentation of Nrf2 activity and may have therapeutic potential for treating COPD [20]. CDDO-Me has been studied for its Nrf2 activation properties and has been deemed a promising drug candidate for treating many different degenerative illnesses, including diabetic complications [21, 22]. A small molecule Nrf2 activator called VEDA-1209 was under preclinical pharmacokinetic and pharmacodynamics testing studies in animal models of ulcerative colitis [23]. Sulforaphane (SFN) is known to induce Nrf2 which plays a central role in chemoprevention and anti-inflammatory. A synthetic sulforaphane-cyclodextrin complex, called Sulforadex, with improved shelf stability over sulforaphane alone has been developed and a first-in-man clinical study of Sulforadex has been completed, and a prostate cancer trial is planned for 2014 [23]. Our present study suggests that PMID shows preventive effect on DSS-induced colitis via inducing Nrf2/ARE activation; it is an interesting question that whether PMID can be developed as a therapeutic agents in treating colitis.

Increasing evidence has shown that Nrf2 could play an important role in defense against oxidative stress possibly by activation of cellular antioxidant machinery as well as suppression of proinflammatory pathways mediated by NF-*κ*B signaling. Interplay between Nrf2 signaling pathway and NF-*κ*B pathway has been observed. NF-*κ*B was shown to prevent the transcription of Nrf2-dependent genes by reducing available coactivator levels and promoting recruitment of a corepressor [24]. Nrf2 has been implicated in NF-*κ*B control through attenuation of phosphorylated I*κ*B, which causes NF-*κ*B degradation [8]. Increased NF-*κ*B activation in* Nrf2* ^−/−^ mice when compared with wild-type after stimuli such as traumatic brain injury [25], LPS [8], TNF*α* [8], ovalbumin [7], and respiratory syncytial virus [26] was observed.* In vivo* and* in vitro* data suggest that many Nrf2 activators confer protective effect against oxidative stress and inflammatory response through suppressing NF-*κ*B signal activation. Ethanol extract of Alismatis Rhizoma reduces LPS-induced acute lung inflammation by suppressing NF-*κ*B and activating Nrf2 [27]. Docosahexaenoic acid protects against inflammation partially via cross talk between Nrf2/heme oxygenase-1 and IKK/NF-*κ*B pathways [28]. The protective effects against methamphetamine-induced neuroinflammation of melatonin result from the inhibition of activated NF-*κ*B in parallel with potentiated antioxidant/detoxificant defense by activated Nrf2 pathway [29]. Schisandrin B exhibits anti-inflammatory activity* in vitro* and* in vivo* through activation of Nrf2 pathway and inhibition of I*κ*B*α* degradation and nuclear translocation of NF-*κ*B [30]. Cyclo (His-Pro) is an endogenous cyclic dipeptide that exerts oxidative damage protection by selectively activating the transcription factor Nrf2 signaling pathway. In a mouse ear inflammation model, Cyclo (His-Pro) was found to reduce 12-otetradecanoylphorbol-13-acetate-induced oedema via suppressing NF-*κ*B signaling and inducing Nrf2-mediated heme oxygenase-1 expression [31]. Our present study suggests the PMID pretreatment decreases the expression levels of inflammatory biomarkers such as TNF*α*, IFN-*γ*, and IL-6 and attenuates the activation of NF-*κ*B with reduced nuclear accumulation of p65, indicating that PMID might also modulate NF-*κ*B pathway* in vivo*. Whether PMID regulates NF-*κ*B pathway depending on induction of Nrf2/ARE pathway needs further confirmation.

In summary, the present study provides evidence that PMID confers protection against DSS-induced colitis in Nrf2-dependent manner* in vivo*.

## Supplementary Material

Figure S1. PMID pre-treatment did not affect the AP1 activity in DSS-induced colon.
Table S1. Disease activity index.
Table S2. Real-time quantitative PCR primers.

## Abbreviations

GAPDH: Glyceraldehyde-3-phosphate dehydrogenase
DSS: Dextran sodium sulphate
PMID: 3-(3-Pyridylmethylidene)-2-indolinone
Nrf2: Nuclear factor (erythroid-derived 2)-like 2
ARE: Antioxidant response element
NQO1: NAD(P)H quinone oxidoreductase-1
HO-1: Heme oxygenase-1
IBD: Inflammatory bowel diseases
NF-*κ*B: Nuclear factor kappa B.
