# Supplementary material for: 3-(3-Pyridylmethylidene)-2-indolinone Reduces the Severity of Colonic Injury in a Murine Model of Experimental Colitis
Source: Oxid Med Cell Longev. 2015 Mar 22;2015:959253. doi: 10.1155/2015/959253 (PMC4385690; doi:10.1155/2015/959253)
Supplement: Supplementary file 1 — Figure S1. PMID pre-treatment did not affect the AP1 activity in DSS-induced colon. Table S1. Disease activity index. Table S2. Real-time quantitative PCR primers. [file 959253.f1.doc]

**Supplementary Figure**


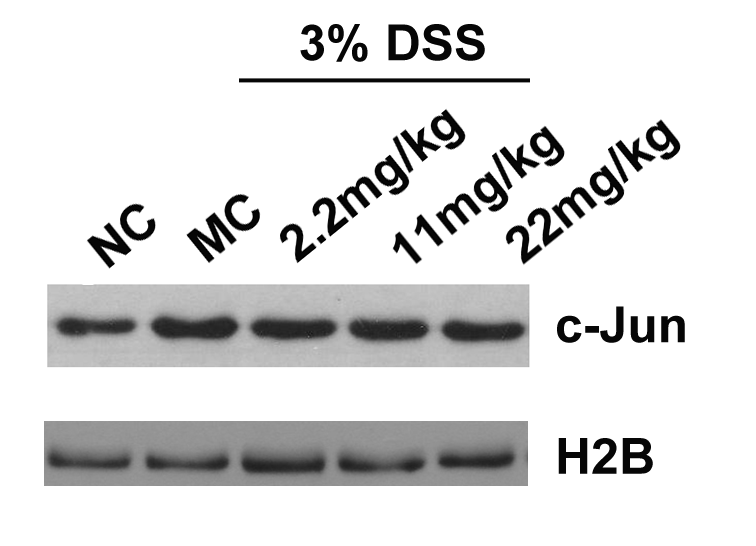


Supplementary Figure 1 PMID pre-treatment did not affect the AP1 activity in DSS-induced colon. Mice were treated with the indicated doses of PMID *per os* for 7 days and then induced to colitis by DSS via drinking water for 7 days. Then the mice were sacrificed and nuclear extracts were prepared for analyzing the protein level of nuclear c-Jun. Histone H2B was used as internal control.

**Supplementary Tables**

**Table S1** Disease activity index

| Score | Weight  loss (%) | Stool*  consistency | Occult/gross  bleeding |
| --- | --- | --- | --- |
| 0 | (-) | Normal | Normal |
| 1 | 1-5 |  |  |
| 2 | 5-10 | Loose | Guiac(+) |
| 3 | 11-15 |  |  |
| 4 | >15 | Diarrhoea | Gross bleeding |

*Normal stools = well formed pellets; loose = pasty stools which do not stick to the anus; diarrhoea = liquid stools that stick to the anus.

Table S2 Real-time quantitative PCR primers

| Gene | Forward primer | Reverse primer |
| --- | --- | --- |
| TNF-α | CCAGACCCTCACACTCAGATC | CACTTGGTGGTTTGCTACGAC |
| IL-6 | CCAGAGATACAAAGAAATGATGG | ACTCCAGAAGACCAGAGGAAAT |
| IFN-γ | GCCACGGCACAGTCATTGA | TGCTGATGGCCTGATTGTCTT |
| HO-1 | CCTCACTGGCAGGAAATCATC | CCTCGTGGAGACGCTTTACAT |
| NQO-1 | TATCCTTCCGAGTCATCTCTAGCA | TCTGCAGCTTCCAGCTTCTTG |
| Nrf2 | CGAGATATACGCAGGAGAGGTAAGA | GCTCGACAATGTTCTCCAGCTT |
| GAPDH | AATGTGTCCGTCGTGGATCT | CATCGAAGGTGGAAGAGTGG |
